# Oxygen level is a critical regulator of human B cell differentiation and IgG class switch recombination

**DOI:** 10.3389/fimmu.2022.1082154

**Published:** 2022-12-14

**Authors:** Jana Koers, Casper Marsman, Juulke Steuten, Simon Tol, Ninotska I. L. Derksen, Anja ten Brinke, S. Marieke van Ham, Theo Rispens

**Affiliations:** ^1^ Department of Immunopathology, and Landsteiner Laboratory, Sanquin Research, Amsterdam University Medical Centers, University of Amsterdam, Amsterdam, Netherlands; ^2^ Department of Research Facilities, and Landsteiner Laboratory, Sanquin Research, Amsterdam University Medical Centers, University of Amsterdam, Amsterdam, Netherlands; ^3^ Swammerdam Institute for Life Sciences, University of Amsterdam, Amsterdam, Netherlands

**Keywords:** B cells, hypoxia, germinal center, differentiation, antibody-secreting cell, class switch recombination

## Abstract

The generation of high-affinity antibodies requires an efficient germinal center (GC) response. As differentiating B cells cycle between GC dark and light zones they encounter different oxygen pressures (*p*O_2_). However, it is essentially unknown if and how variations in *p*O_2_ affect B cell differentiation, in particular for humans. Using optimized *in vitro* cultures together with in-depth assessment of B cell phenotype and signaling pathways, we show that oxygen is a critical regulator of human naive B cell differentiation and class switch recombination. Normoxia promotes differentiation into functional antibody secreting cells, while a population of CD27^++^ B cells was uniquely generated under hypoxia. Moreover, time-dependent transitions between hypoxic and normoxic *p*O_2_ during culture - reminiscent of *in vivo* GC cyclic re-entry - steer different human B cell differentiation trajectories and IgG class switch recombination. Taken together, we identified multiple mechanisms trough which oxygen pressure governs human B cell differentiation.

## Introduction

Development of an effective long-lasting immune response implies development of high-affinity, class-switched antibodies, the result of B cell differentiation within so-called germinal centers (GC), specialized substructures in secondary lymphoid tissues. Upon initiation of the humoral immune responses, initial T cell-dependent B cell activation leads to generation of a GC response, which is preceded by a short period of extrafollicular B cell activation. During the initial extrafollicular response, an early wave of naive B cells differentiate into plasma blasts and early memory B cells. These cells display limited levels of somatic hypermutation and affinity maturation (1–4). The GC forms approximately one week after antigen exposure and becomes organized into two histologically distinct regions; the dark zone, where B cells undergo extensive proliferation and somatic hypermutation, and the light zone, where high-affinity B cells compete for antigen in order to undergo affinity selection. Class switch recombination may occur as early as the pre-GC stages and ensues in the GC reactions (5–7). GC B cells cycle repeatedly through the light zone and dark zone. After initial fate decision, differentiation may proceed towards affinity-matured late memory B cells or antibody secreting cells (i.e., plasmablasts and plasma cells) with accumulating affinity maturation (8–10).

Previous studies by ourselves and others using *in vitro* cultures of human or mouse B cells demonstrated that a GC-like B cell response can be faithfully reproduced, including generation of mature memory B cells and antibody secreting cells (11, 12). Similar to what has been reported *in vivo*, CD40 ligation together with the availability of Tfh-associated cytokines IL-4 and IL-21 is required to promote B cell proliferation, isotype switching and generation of antibody secreting cell *in vitro* (2, 13–15). The combined action of Tfh-derived CD40L, IL-21, and IL-4, results in activation of NFκB and JAK-STAT pathways. These are vital for, amongst others, upregulation of BLIMP1 and XBP-1s, transcription factors necessary for initiation of antibody secreting cell differentiation and antibody production (16–20), as well as C-Myc and IRF4 upregulation (18, 21–23). C-Myc is required for the survival of GC B cells and is strongly upregulated upon antigen-specific selection in the GC light zone, allowing for dark zone (re-) entry (24–27). A central role in orchestrating these events is the transcriptional repressor BCL6, which regulates expression of amongst others c-Myc and BLIMP1 (24, 28, 29) (For more detail, see **Figure S4A**).

The partial pressure of oxygen (*p*O_2_) in healthy human tissues is around 3-6% (30, 31), but within lymphoid tissues, distinctive hypoxic regions exist (*p*O_2_ ~0.5-1%), as specifically observed in GC light zone regions (32–34). However, the role of variations in *p*O_2_ on human B cell differentiation, and fate decision into memory B cells and antibody secreting cells has not been studied. In fact, the vast majority of *in vitro* studies is carried out at atmospheric oxygen levels ((*p*O_2_ ~21%), which is much higher than proliferating B cells will encounter *in vivo*.

Variations in *p*O_2_ are likely to affect the amplitude of B cell differentiation, due to profound effects of cellular metabolism as well as direct effects on transcriptional regulation (30, 31, 35–38). Although, contrasting findings have been reported for the influence of hypoxia on GC responses in mice, negative effects on class switch recombination and proliferation are repeatedly described (32, 34, 39). Others observed increased Tfh function induced by upregulation of HIF-1α, a major transcription factor involved in the cellular sensing of *p*O_2_ (33). Not all previous studies have been able to confirm HIF-1α expression within GC B cells, suggesting hypoxia may regulate B cell function independent of HIF-1α (40). Another study comparing hypoxic (1%) and venous (5%) *p*O_2_ reported decreased class switch recombination and altered cellular metabolism upon culture at hypoxic *p*O_2_ (32). Overall, oxygen pressure appears an important but as yet poorly understood variable in B cell differentiation.

In the present study, we systematically investigated the effect of atmospheric (21%), normoxic, tissue-associated (3%), and hypoxic (1%) *p*O_2_ on human B cell differentiation in our highly optimized system for human primary B cell culture. Moreover, in line with varying oxygen pressures during B cell cycling in the GC dark zone and light zone, we studied time-dependent transitions in *p*O_2_ during culture and its effects on B cell differentiation trajectories and IgG class switch recombination. We show that different oxygen pressures distinctly regulate human naive B cell differentiation and class switch recombination.

## Results

### Differential *p*O_2_ controls human B cell differentiation into CD27^+^ and antibody secreting cell compartments

**Figure 1:**
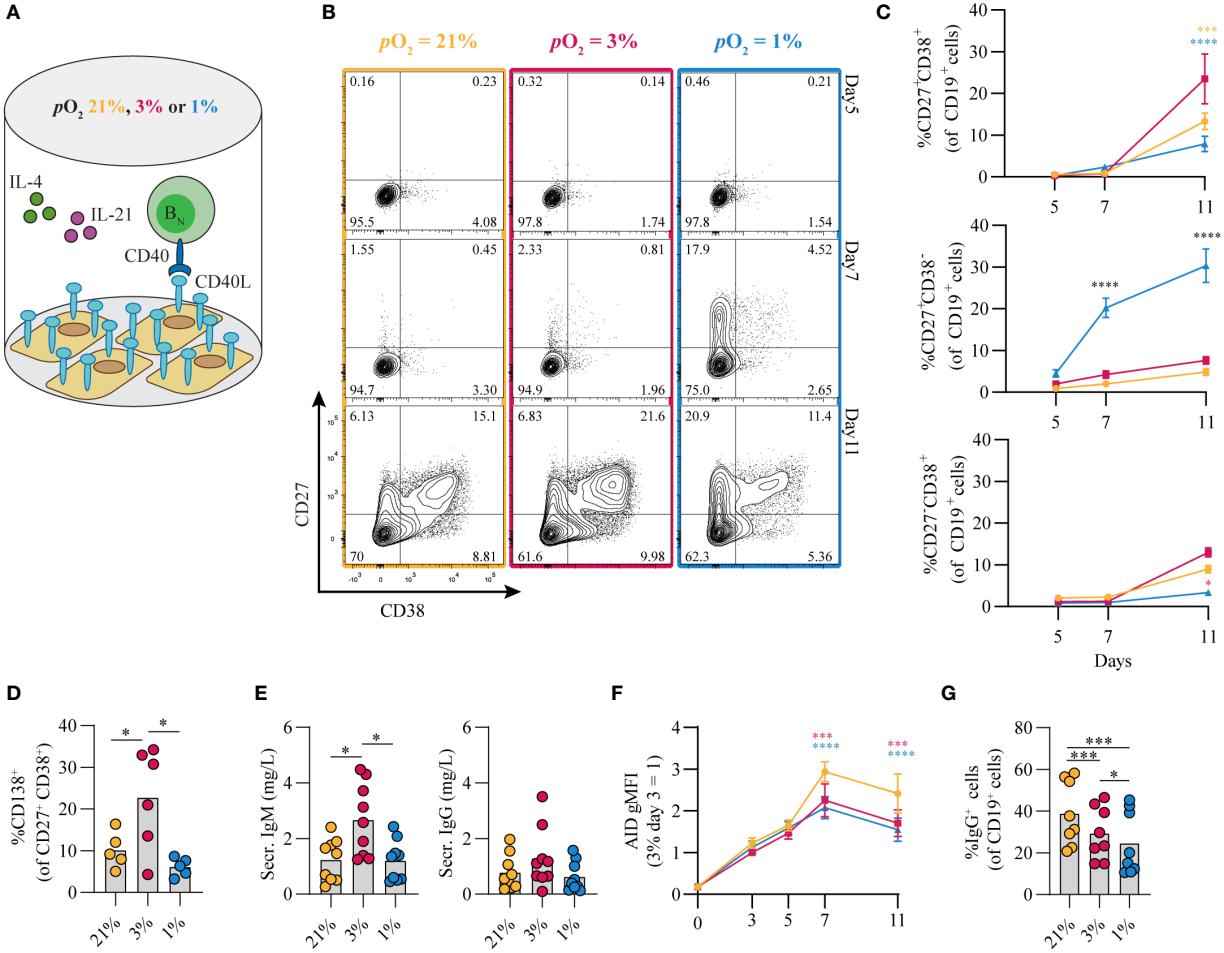
Differential *p*O_2_ controls human B cell differentiation into CD27^+^ and antibody secreting cell compartments. **(A)** Schematic overview of B cell *in vitro* culture system. 250 resting human naive B cells (CD19^+^CD27^-^IgD^+^) were stimulated using a human CD40L-expressing feeder cell layer (subtype ‘High’ (12),); recombinant human IL-4 (25ng/ml) and IL-21 (50ng/ml); and cultured at 5% *p*CO_2_ and 21, 3, or 1% *p*O_2_ for a maximum of 11 days. **(B)** Representative biaxial CD27/CD38 FACS plots after 5, 7, and 11 days of culture at 21, 3, or 1% *p*O_2_. **(C)** Quantification of the percentages of CD27 and CD38 subpopulations within total CD19^+^ B cells over time (*n* = 9). **(D)** CD138 expression within the CD27^+^CD38^+^ antibody secreting cell population (*n* = 6). **(E)** Cumulative secretion of IgM and IgG measured in culture supernatants after 11 days (*n* = 9). **(F)** gMFI of AID expression over time (*n* = 3). **(G)** Percentage of IgG^+^ cells within CD19^+^ B cells, combined surface and intracellular staining (*n* = 8). Bars represent means of biological replicates each composed of 2 technical replicates in **(D, F)** 2, **(E, G)** 3 or **(C)** 4 independent experiments. Statistical differences were determined using **(C, F)** mixed-effects analysis using Tukey’s test for multiple comparisons **(D, E, G)** repeated measures one-way ANOVA using Tukey’s test for multiple comparisons. *p < 0.05, ***p < 0.001, ****p < 0.0001.

The contribution of *p*O_2_ on human naive B cell differentiation was investigated in B cell differentiation cultures while maintaining constant *p*CO_2_. Resting naive B cells (CD19^+^IgD^+^CD27^-^IgG^-^IgA^-^) from human peripheral blood were stimulated under atmospheric (*p*O_2_ = 21%), normoxic (*p*O_2_ = 3%) or hypoxic (*p*O_2_ = 1%) conditions, using a combination of CD40L-expressing 3T3 cells (12), IL-4 and IL-21, known to facilitate class switch recombination, memory B cell and antibody secreting cell formation (**Figure 1A**) (12, 41, 42). Alterations in *p*O_2_ did not affect CD40L expression levels on 3T3 cells used for stimulation (**Figure S2I**, subtype high). Up until day 7 B cell survival was similar among *p*O_2_ conditions, but at day 11 hypoxic cultures contained fewer cells (**Figures S2A,B**). In all cultures a rapid upregulation of activation marker CD80 was observed (**Figure S2C**). Remarkably, naive B cell differentiation, as determined by the formation of antibody secreting cells (CD27^+^CD38^+^) and CD27^+^ (CD27^+^CD38^-^) B cells, was dramatically altered by differential *p*O_2_. Hypoxia induced a 5 to 10-fold induction of CD27^+^ B cells as compared to atmospheric (p<0.0001) and normoxic cultures (p<0.0001) at day 7 (**Figures 1B, C**), and absolute cell no. in **Figure S2D**), at the expense of antibody secreting cell formation at day 11. In addition, in normoxic cultures, the relative numbers of CD138^+^ antibody secreting cells were 2 to 4-fold increased relative to atmospheric (p<0.001) and hypoxic cultures (p<0.0001, **Figure 1D**), in line with increased concentration of secreted IgM and IgG in normoxic culture supernatant at day 11 (**Figure 1E**). Naive B cells poorly survived and differentiated when cultured under extreme hypoxic conditions (*p*O_2_ = 0.5%, **Figure S2E, F**). Furthermore, using 3T3 cells with higher or lower expression of CD40L resulted in reduced proliferation, especially at reduced oxygen pressures (resp. ‘VH’, and ‘Low’, vs ‘High’; **Figures S2H, I**). The addition of anti-IgM F(ab’)_2_s to the cultures, as additional BCR stimulation, did not alter *in vitro* B cell survival and differentiation at differential *p*O_2_s (**Figure S2J**). Next, we assessed the effect of *p*O_2_ on antibody class switching and Ig production. Protein expression of DNA-editing enzyme AID –indispensable for class switch recombination and somatic hypermutation– were substantially elevated over time regardless of oxygen pressure, but significantly lower at low *p*O_2_ at day 7 and 11 (**Figure 1F**), in accordance with previous studies in mice (32), reflecting the overall larger fraction of differentiated cells under these conditions (**Figures 1B, C**,**S2K**). In line with AID expression levels, IgG^+^ B cell frequencies were higher in atmospheric cultures (p<0.001, **Figure 1F**) with minimal switch to IgA^+^ overall, as was expected by the IgG promoting culture conditions (CD40L + IL-21 + IL-4, **Figure S2L**). Altogether, these data illustrate a profound regulation of human naive B cell differentiation by oxygen levels and show that normoxia promotes differentiation into potent antibody secreting cells while hypoxia promotes differentiation into CD27^+^ B cells.

### Proliferative human B cells adopt glycolysis and mitochondrial-associated ROS levels are increased in hypoxic cultures

**Figure 2:**
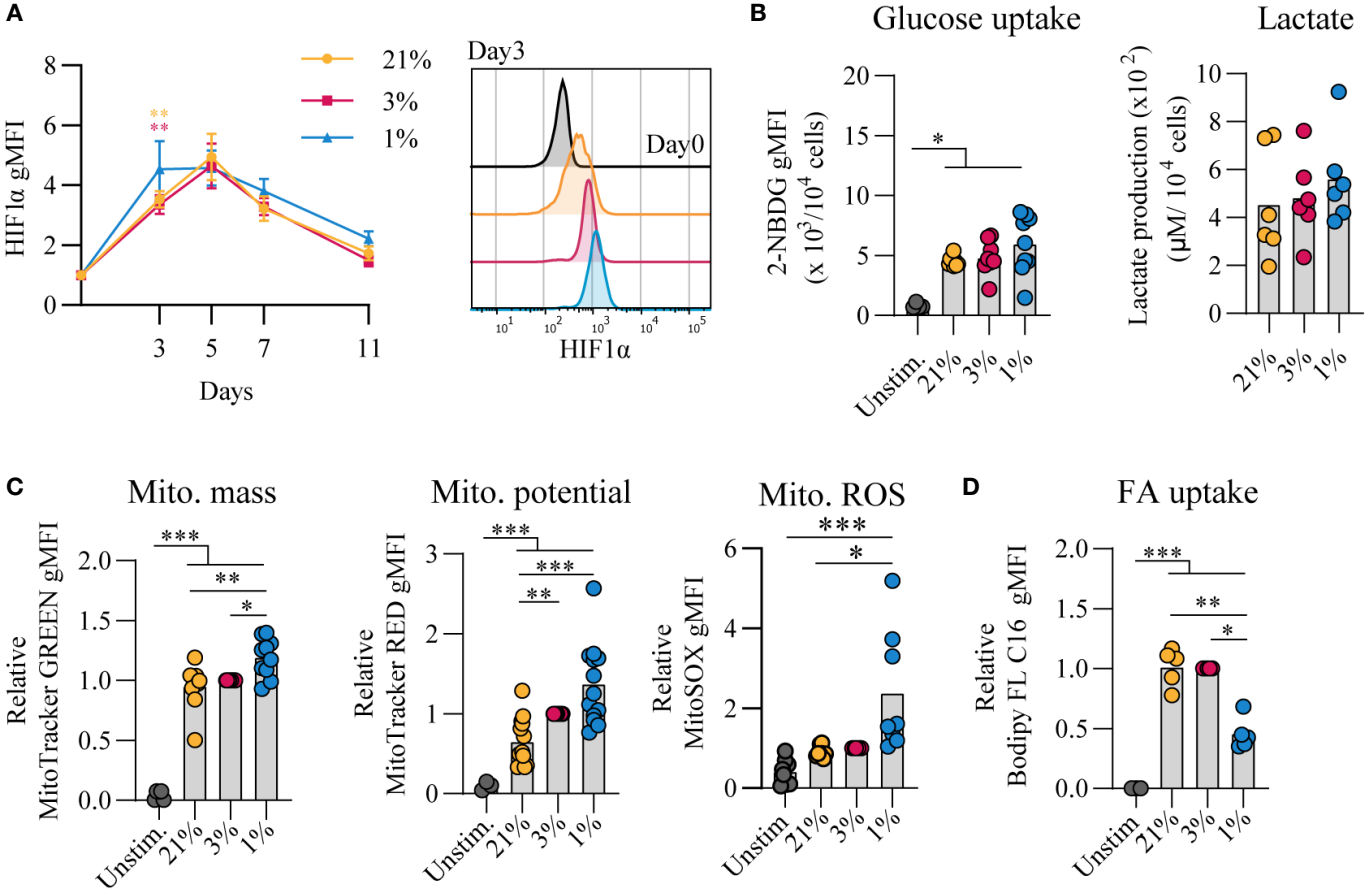
Proliferative human B cells adopt glycolysis and mitochondrial-associated ROS levels are increased in hypoxic cultures **(A)** gMFI of HIF1α expression over time (*n* = 6) and histogram overlay at day 3. **(B)** Glucose uptake (2-NBG; (2-(N-(7-nitrobenz-2-oxa-1,3-diazol-4-yl)amino)-2-deoxyglucose) by B cells cultured for 7 days (*n* = 9). Lactate production measured in supernatants at day 11 in µM per 10^4^ cultured B cells (*n* = 6). **(C)** gMFI of MitoTracker GREEN, MitoTracker RED, and MitoSOX after 7 days of culture (*n* = 8) indicative for mitochondrial mass, potential and ROS production, respectively. **(D)** Fatty acid (FA) uptake by B cells cultured for 7 days (*n* = 5). Bars represent means of biological replicates each composed of two technical replicates of **(E)** 1, **(A, C)** 2 or **(B, D)** 3 independent experiments. Statistical differences were determined using a repeated measures one-way ANOVA using Tukey’s test for multiple comparisons. *p < 0.05, **p < 0.01, ***p < 0.001.

Given that cellular metabolism has been reported to be linked to gene expression and B cell differentiation (43), we assessed the metabolic status of B cells that were cultured at different *p*O_2_s. Using flow cytometry, HIF1α levels were increased throughout hypoxic cultures compared to normoxic and atmospheric cultures (**Figure 2A**). After 7 days of *in vitro* stimulation B cells showed similar increase in cell size in all conditions compared to unstimulated cells (**Figure S3A**). Dividing lymphocytes typically rely on aerobic glycolysis, fermenting imported glucose into lactate rather than oxidizing it in the mitochondria for energy. Glycolytic activity was assessed by uptake of 2-NBDG, a fluorescent analog of glucose, and production of lactate as an end product of glycolysis. Uptake of 2-NBDG and lactate production were not significantly different between different *p*O_2_ culture conditions, but overall higher compared to resting naive B cells suggesting that indeed stimulated B cells require more glucose but demands did not change considerably upon differential *p*O_2_ environments (**Figure 2B**). Also expression of GLUT1, a glucose transporter, varied minimally between culture conditions (**Figure S3B**). To asses mitochondrial oxidative metabolism we analyzed mitochondrial mass, potential and formation of reactive oxygen species (ROS) by flow cytometry at day 7. Mitochondrial mass, potential and mitochondrial ROS were higher in B cells cultured at hypoxic conditions (**Figure 2C**). Uptake of a fluorescent fatty acid probe was reduced in B cells under hypoxic conditions (**Figure 2D**), suggesting these cells rely less on oxidation of fatty acids. At day 11 metabolic markers yielded similar result among different *p*O_2_ conditions, except for ROS levels that were significantly higher in hypoxic cultures compared to atmospheric cultures (**Figure S3C**). Taken together, these results indicate that highly proliferative B cells adopt glycolysis independent of local *p*O_2_ levels and mitochondrial-associated ROS levels are elevated in hypoxic cultures.

### *p*O_2_ steers canonical signaling pathways that direct naive B cell differentiation pathways

**Figure 3:**
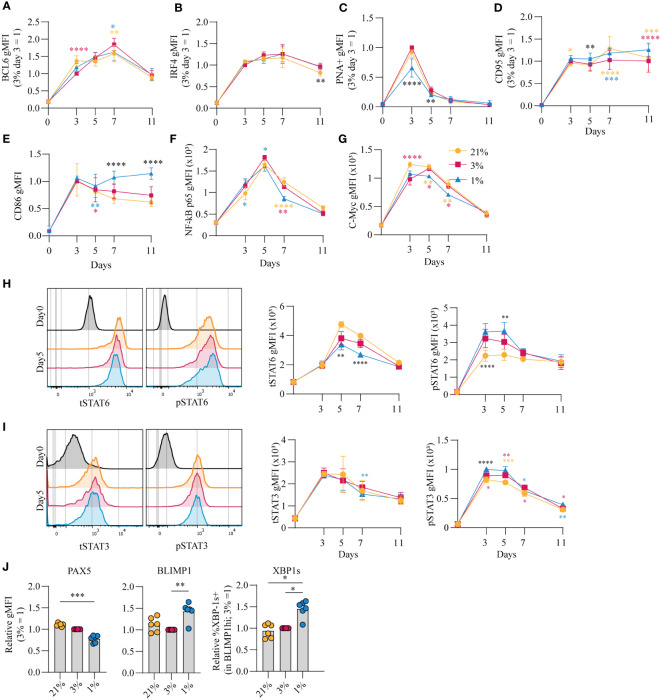
pO_2_ steers canonical signaling pathways that direct naive B cell differentiation pathways **(A-D)** gMFI of **(A)** BCL-6 (*n* = 6) **(B)** IRF4 (*n* = 6) **(C)** PNA+ (*n* = 6) **(D)** CD95 (*n* = 6) **(E)** CD86 (*n* = 6) **(F)** NFκB active subunit p65 (*n* = 9) **(G)** c-Myc (*n* = 9) **(H-I)** gMFI of **(H)** tSTAT6 and pSTAT6 and **(I)** tSTAT3 and pSTAT3 over time in culture (*n* = 6) and (left panels) representative histogram overlays of pSTAT and tSTAT expression on day 5 of culture **(J)** gMFI of PAX5, BLIMP1 and %XBP-1s+ in BLIMP1hi cells (n=6) on day 7 **(A-J)** Bars represent means of biological replicates each composed of two technical replicates of 2 **(H-J)** or **(A-G)** 3 independent experiments **(A-I)** Differences in gMFI were determined using mixed-effects analysis using Tukey’s test for multiple comparisons. **(J)** Differences in gMFI were determined using repeated measures one-way ANOVA using Tukey’s tests for multiple comparisons. *p < 0.05, **p < 0.01, ***p < 0.001, ****p < 0.0001.

The amplitude of B cell proliferation and differentiation is regulated by a complex interplay of signaling pathways induced by antigen recognition, CD40 ligation and reception of Tfh-derived cytokines IL-4 and IL-21 (summarized in **Figure S4A**) (8, 44). Upregulation of BCL6 as well as IRF4 expression suggests differentiation of cells with a GC-like phenotype (**Figures 3A, B**, respectively). In line, PNA positivity, indicative for expression of GC-specific glycans, and CD95 expression was observed early on in culture and decreased prior to the generation of CD27^+^ B cells and antibody secreting cells (**Figures 3C, D**). PNA binding was reduced for B cells cultured at hypoxic *p*O_2_ at day 3 and 5 potentially due to the accelerated CD27^+^ differentiation in hypoxic cultures. Expression of CD86, an activation marker highly expressed on light zone B cells (45, 46), increased rapidly during culture and remained significantly higher expressed on B cells cultured at hypoxic *p*O_2_ (**Figure 3E**). CD40 signaling primarily shapes the magnitude of B cell expansion and survival through induction of NFκB p65 and c-Myc (21–23). Moreover, NFκB p65 and c-Myc are reported to maintain cellular GC commitment (47). B cells cultured at hypoxic *p*O_2_ showed a reduction in NFκB p65 (**Figure 3F**) and c-Myc (**Figure 3G**) expression from day 5 onwards, supporting the concomitant differentiation into CD27^+^ cells, and to a lesser extent antibody secreting cells, at day 7 and reduced B cells numbers observed in hypoxic cultures thereafter (**Figure S2B**).

Next, the effect of *p*O_2_ on the IL-4R/IL-21R signaling pathways to regulate B cell and antibody secreting cell transcriptional programs was analyzed. Increased phosphorylation of STAT6 (pSTAT6), a signaling protein downstream of the IL-4R, was observed in B cells cultured at hypoxic *p*O_2_, peaking at day 3 and 5 (p<0.01 at day 5) (**Figure 3H**, **S4B**). This coincided with higher expression of XBP-1s, a transcription factor (TF) downstream of pSTAT6, at day 7 (**Figure 3J**, right panel), and was not the result of variable IL-4R expression levels, determined by RT-qPCR (**Figure S4C**). Peak levels of pSTAT3 on day 3 and 5, with minor differences when comparing the different pO_2_ conditions (**Figure 3I**, **S4D**), coincided with upregulation of BLIMP1, directly downstream of pSTAT3, and repression of PAX5, most prominently in B cells cultured at hypoxic pO_2_ (**Figure 3J**, **S4E**). PAX5 supports B cell identity. Consistent with this activity, B cells cultured at atmospheric pO_2_ retained highest PAX5 levels throughout culture, and thus overall a lower fraction of differentiated cells under this conditions (**Figure 3I**, **S4D**) (48). Expression of BLIMP1 and XBP-1s was higher under hypoxic conditions (**Figure 3J**), and did not differ within the CD27^-^CD38^-^ population at varying *p*O_2_ at day 7 (**Figure S4F**), suggesting elevated BLIMP1 and XBP-1s expression in B cells cultured at hypoxic *p*O_2_ arises from the expanded CD27^+^ compartment (**Figure 1C**). This CD27^+^ population did not secrete more Ig at day 7 compared to cells cultured at normoxic or atmospheric *p*O_2_ despite elevated XBP-1s expression, which is essential for the unfolded protein response in antibody secreting cells (**Figure S4G**). At day 11, naive B cells differentiation into antibody secreting cells in normoxic cultures outperformed atmospheric cultures in which B cells retained higher PAX5 levels and repression of BLIMP1 and XBP-1s (**Figure S4H**). Furthermore, cells in normoxic cultures had highest XBP-1s levels in line with higher Ig production observed in these cultures (p<0.05) (**Figure 1G**). Overall, B cell culture at different *p*O_2_ affects CD40-, IL-21R- and IL-4R-dependent signaling pathways essential for efficient B cell survival and differentiation. Expression dynamics of B differentiation-associated TFs PAX5, BLIMP1 and XBP1 were influenced by local *p*O_2_, skewing the formation of CD27^+^ and/or antibody secreting cell populations at hypoxic or normoxic *p*O_2_, respectively.

### Hypoxic *p*O_2_ drives generation of a unique CD27^++^ B cell population, with enhanced antibody secreting cell differentiation capacity and Ig production upon restimulation

**Figure 4:**
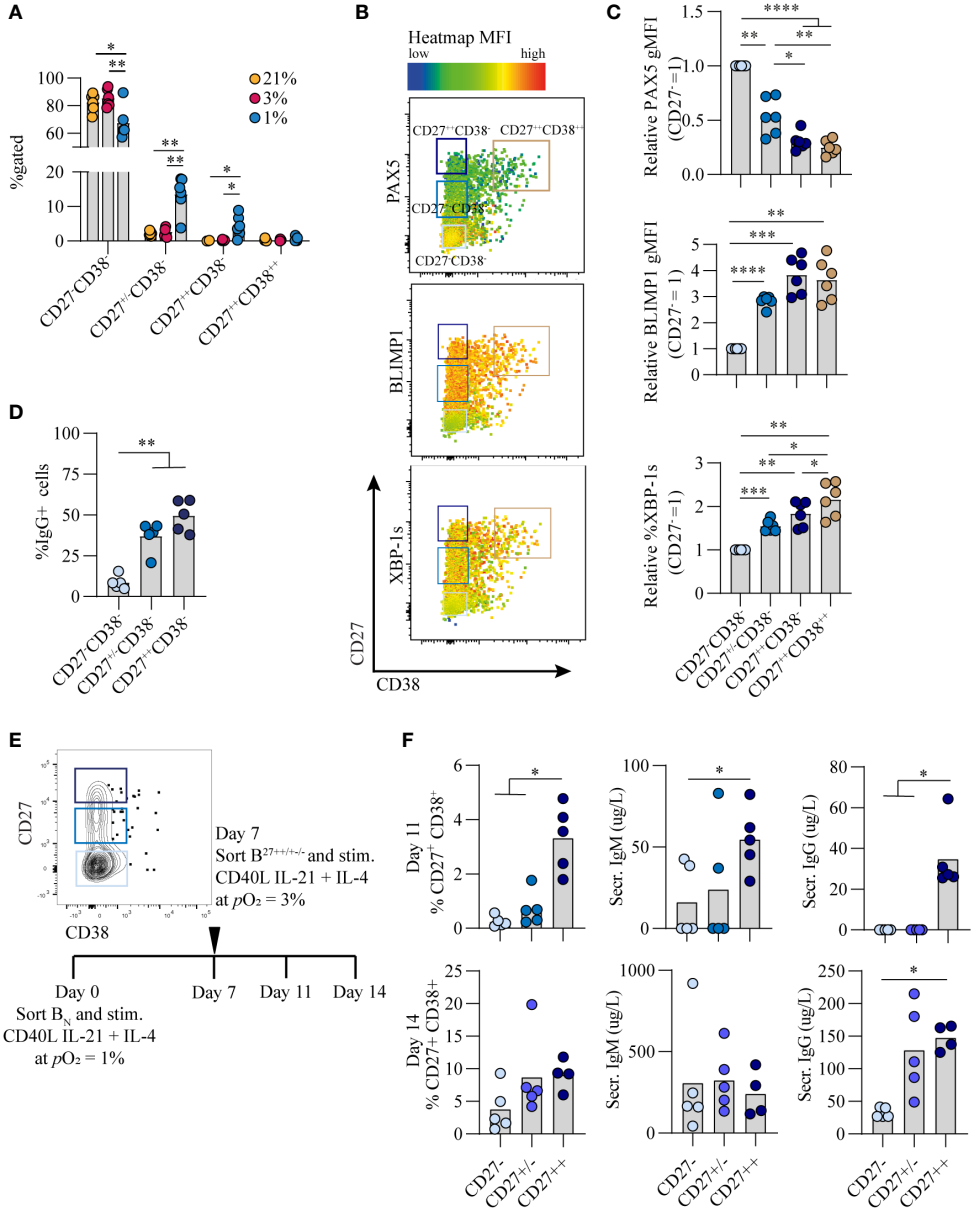
Hypoxic *p*O_2_ drives generation of a unique CD27^++^ B cell population, with enhanced antibody secreting cell differentiation capacity and Ig production upon restimulation **(A)** Quantification of percentage of CD27^-^CD38^-^, CD27^+/-^CD38^-^, CD27^++^CD38^-^ and CD27^++^CD38^++^ cells on day 7 of culture (*n* = 5) **(B)** Representative biaxial flow cytometry plot of CD27 and CD38 expression, with heatmap depicting MFI expression of PAX5, BLIMP1 and XBP-1s on day 7. **(C)** gMFI of PAX5, BLIMP1 and XBP-1s in respective populations of CD27^-^CD38^-^, CD27^+/-^CD38^-^, CD27^++^CD38^-^ and CD27^++^CD38^++^ cells on day 7 (*n* = 6) **(D)** Percentage of IgG+ B cells on day 7 of of culture at 1% pO_2_ within CD27^++^ CD38^-^, CD27^+/-^ CD38^-^ or CD27^-^ CD38^-^ populations. (*n* = 5) **(E)** Representative example of FACS sort gating strategy on day 7 to isolate CD27^++^ CD38^-^, CD27^+/-^ CD38^-^ or CD27^-^ CD38^-^ B cells with schematic experimental time line. **(F)** Quantification of %CD27^+^CD38^+^ cells by flow cytometry and IgG and IgM secretion by ELISA, 4 or 7 days after sort (day 11 or 14 of culture) (*n* = 5). **(A-F)** Bars represent means of biological replicates each composed of two technical replicates of **(A, C, D)** 2 or **(F)** 1 independent experiment **(A)** Differences in %gated cells were determined using mixed-effects analysis using Tukey’s test for multiple comparisons. **(C, D, F)** Differences in gMFI and % gated cells were determined using repeated measures one-way ANOVA using Tukey’s test for multiple comparisons. *p < 0.05, **p < 0.01, ***p < 0.001, ****p < 0.0001.

Culturing B cells at hypoxic *p*O_2_ resulted in a remarkable increase in CD27^+^ B cells which emerged earlier as compared to atmospheric and normoxic cultures (**Figure 1C**). Interestingly, there was not only enlargement of this compartment but also formation of a population of CD27^++^ cells (4.4% of CD19+ population), absent in normoxic and atmospheric cultures at day 7 (p<0.05) (**Figure 4A**, **S5**) To assess the phenotype of these CD27^++^ B cells, transcription factor profiles directing B cell differentiation were compared between CD27^-^, CD27^+/-^, CD27^+/+^ and CD27^++^CD38^++^ populations that developed under hypoxic *p*O_2_ at day 7 (**Figure 4B**). PAX5 expression was lower and BLIMP1 expression higher in CD27^+/++^ cells compared to CD27^-^CD38^-^ B cells, with PAX5 and BLIMP1 expression being similar between CD27^++^ and CD27^++^CD38^++^ populations (**Figure 4C**, upper and middle panel). Expression of XBP-1s increased as differentiation progressed, being highest in the CD27^++^CD38^++^ population (**Figure 4C**, lower panel). Moreover, a rise in the frequency of IgG^+^ B cells coincided with increasing CD27 expression, being highest in the CD27^++^ population (**Figure 4D**).

As the CD27^++^ showed a distinct transcriptional profile compared to CD27^+/-^ and CD27^-^ B cells, we assessed whether this translated into functional differences with regard to antibody secreting cell differentiation and Ig production. To this end naive B cells were cultured for 7 days under hypoxia, after which different CD27 expressing populations were sorted and subsequently cultured at antibody secreting cell-promoting normoxic *p*O_2_ for an additional 4 or 7 days (**Figure 4E**). CD27^++^ cells showed a significant increase in antibody secreting cell differentiation and antibody production as compared to the CD27^+/-^ and CD27^-^ subsets (p<0.05) (**Figure 4F**), indicating that with the same level of stimulation the CD27^++^ cells had enhanced capacity to differentiate towards functional antibody secreting cells. In summary, a population of CD27^++^ B cells emerged exclusively in hypoxic cultures, with the potential to rapidly differentiate into antibody secreting cells and produce Ig after restimulation.

### Time-dependent *p*O_2_ transitions alter B cell differentiation dynamics and promote class switch recombination to IgG

**Figure 5:**
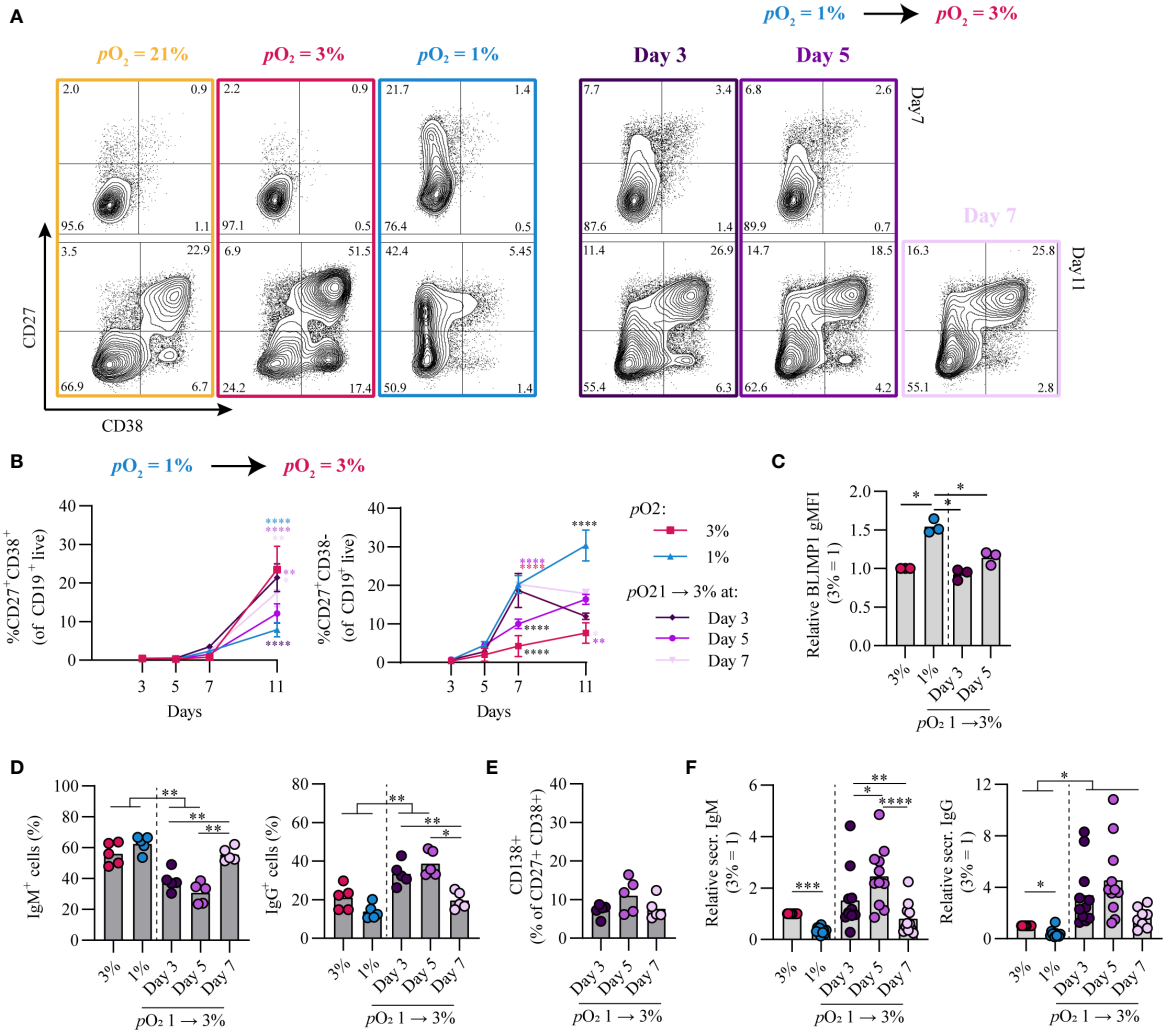
Time-dependent pO_2_ transitions alter naive B cell differentiation and IgG class switch recombination. **(A)** Representative biaxial CD27/CD38 FACS plots of 21, 3, 1% pO2 cultures and 1 – 3% pO_2_ transition cultures at day 3, 5 or 7 shown for day 7 and 11 of culture. **(B)** Quantification of CD27^+^CD38^+^ and CD27^+^CD38^-^ B cells over time in culture (*n* = 11) **(C)** gMFI of BLIMP1 on day 7 (*n* = 3) **(D)** Frequency of IgM^+^ and IgG^+^ B cells on day 11 (*n* = 5). **(E)** CD138 expression within the CD27^+^CD38^+^ population (*n* = 5). **(F)** IgM and IgG levels were measured by ELISA in culture supernantant after 11 days (*n* = 11). Bars represent means of biological replicates each composed of two technical replicates of **(B, F)** 3, **(D)** 2 or **(C, E)** 1 independent experiment. **(B)** Statistical differences were determined using mixed-effects analysis using Tukey’s test for multiple comparisons **(C-F)** Differences in gMFI, % gated cells and Ig production were determined using repeated measures one-way ANOVA using Tukey’s test for multiple comparisons. *p < 0.05, **p < 0.01, ***p < 0.001, ****p < 0.0001.

Given that during an *in vivo* GC reaction B cells cycle between the hypoxic light zone and normoxic dark zone, we assessed the effect of a *p*O_2_ transition on B cell differentiation and class switch recombination *in vitro*. To this end, naive B cells were cultured at either hypoxic or normoxic *p*O_2_ and transferred at stated time points to normoxic or hypoxic *p*O_2,_ respectively. Independent of timing, a transition from hypoxic to normoxic *p*O_2_ increased antibody secreting cell differentiation concomitant with decreased CD27^+^ cell formation (**Figures 5A, B**). Transition from hypoxic to normoxic *p*O_2_ at day 3 of culture enhanced antibody secreting cell differentiation at day 11 compared to continued culture at hypoxic pO_2_ and was similar to continuous culture under normoxic *p*O_2_ (**Figures 5A, B**), with BLIMP1 expression lower compared to hypoxic cultures and similar to levels observed in normoxic cultures (**Figure 5C**). A transition from 1 to 3% *p*O_2_ at day 5 led to a moderate generation of CD27^+^ B cells and reduced antibody secreting cells formation in line with continuous hypoxic cultures. When hypoxic cultures were transitioned to normoxic pO_2_ at day 7, this induced an efficient formation of antibody secreting cells at day 11. Different from cultures at hypoxic *p*O_2_ on day 3 and 5, at day 7 hypoxic cultures consist for a significant proportion (~20%) of CD27^+/++^ B cells, which efficiently differentiate to antibody secreting cells upon restimulation and transition to normoxic *p*O_2_ as was shown in **Figure 4F**. Overall this suggests time-dependent transitions in *p*O_2_ steer B cell differentiation, where early (day 3) transitions from hypoxic to normoxic *p*O_2_ lead to antibody secreting cell formation comparable to continuous normoxic cultures whereas a *p*O_2_ transition later on during culture (day 7) induces antibody secreting cell formation - in part - *via* the CD27^++^ compartment. These data show that *in vitro* B cell differentiation can occur *via* distinct trajectories steered by differential *p*O_2_, where hypoxia drives differentiation *via* a CD27^++^ population and atmospheric and normoxic *p*O_2_ *via* CD38^+^ or directly towards antibody secreting cells (**Figure S6A**). Remarkably, transitions from hypoxic to normoxic *p*O_2_ at day 3 and 5 enhanced class switch recombination to IgG^+^ B cells concomitant with a reduction in IgM^+^ B cells (**Figure 5D**). Even though the percentage of CD138^+^ antibody secreting cells was similar (**Figure 5E**) and lower compared to continuous normoxic cultures (**Figure 1D**), transition of hypoxic to normoxic *p*O_2_ at day 3 and 5 resulted in higher quantities of secreted IgM and IgG in culture supernatants (**Figure 5F**). Enhanced IgG class switch recombination and Ig production were no longer observed when the *p*O_2_ transition was made at day 7.

Vice versa, transition from normoxic to hypoxic *p*O_2_ at day 3 and 5 enhanced CD27^+^ B cell formation and BLIMP1 expression compared to continuous normoxic cultures and was more similar to continuous hypoxic cultures (**Figures S6B–D**). A transition at day 7 from normoxic to hypoxic *p*O_2_ was deleterious for both CD27^+^ B cell and antibody secreting cell differentiation. class switch recombination to IgG^+^ B cells and Ig production levels ranged between those found in continuous normoxic and hypoxic cultures (**Figures S6E–G**).

Overall, time-dependent transitions between hypoxic and normoxic *p*O_2_ during culture govern different human B cell differentiation trajectories and IgG class switch recombination *in vitro*.

### Discussion

**Figure 6:**
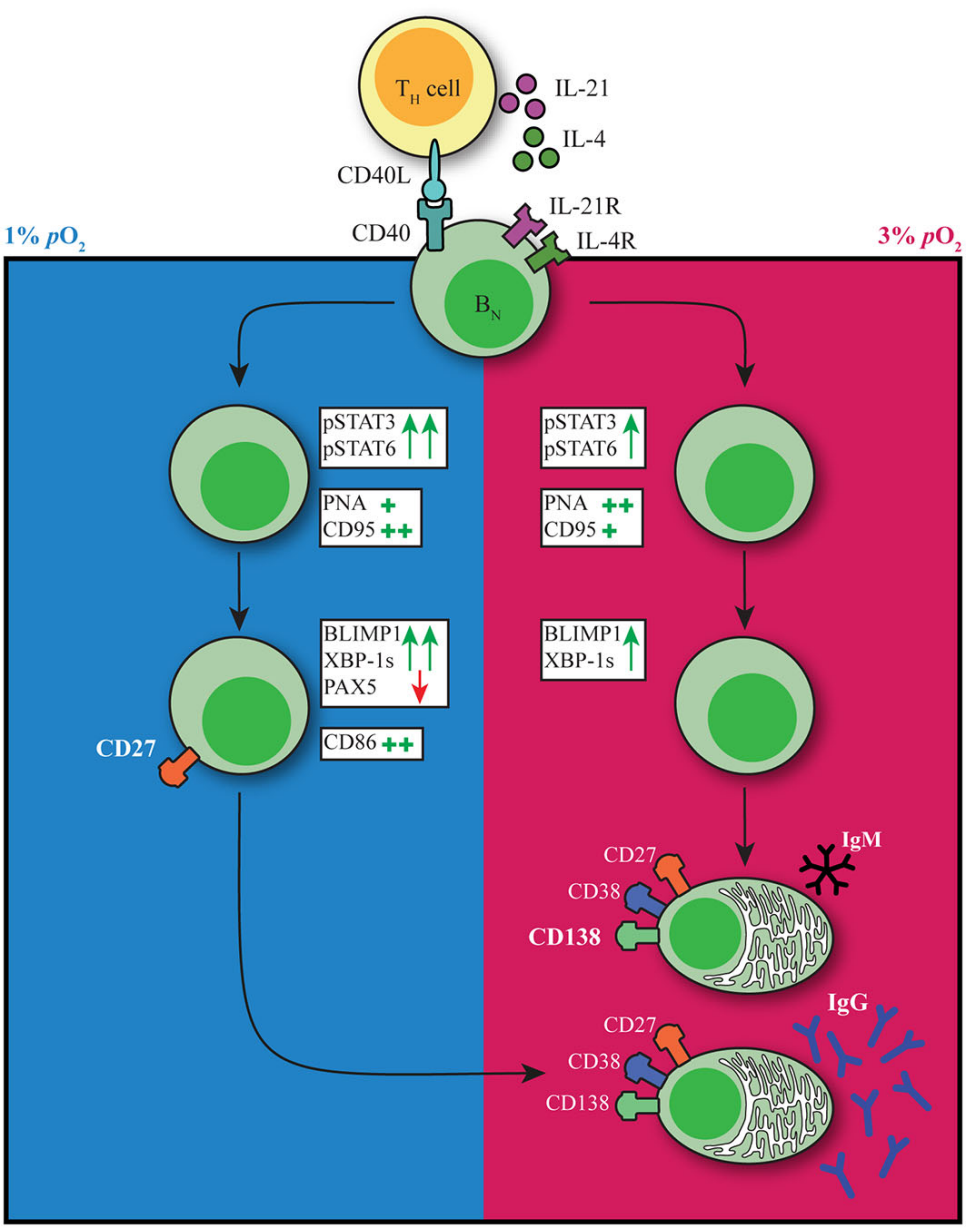
pO_2_ as a critical driver of B cell differentiation *in vitro*. Schematic representation of the effect of differential *p*O_2_ on B cell differentiation and underlying signaling pathways.

The GC reaction is at the heart of the humoral immune response. Both extrafollicular and GC B cell responses take place within human tissues where oxygen pressure may range from 0.5-6% *p*O_2_ with hypoxic regions (0.5-1% *p*O_2_) described within GC light zone regions. Preciously little information is available about the potential role of local *p*O_2_ on human B cell fate decision during their cycling between dark zone and light zone, typically characterized by dissimilar oxygen pressures. Here we studied the effect of atmospheric (21%), tissue associated (normoxia, 3%) and hypoxic (1%) *p*O_2_ on human B cell differentiation. We observe profound differences in B cell differentiation, dynamics of emergence of various cell populations, class switch, and Ig production under these different oxygen pressures. **Figure 6** summarizes our key findings on the contribution of *p*O_2_ on human naive B cell differentiation. In both hypoxic and normoxic cultures, naive B cells differentiated into a GC-like phenotype as evidenced by the expression of BCL6 and IRF4, as well as increased expression of CD95 and PNA binding. Similar to light zone B cell phenotype and function *in vivo* (8), B cells cultured at hypoxic *p*O_2_ showed high expression of CD86 and differentiated rapidly into CD27^+^ B cells. Moreover, culture at hypoxic *p*O_2_ generated a unique population of CD27^++^ B cells efficient in antibody secreting cell differentiation and Ig production upon restimulation. In normoxic cultures, naive B cells predominantly differentiated into antibody secreting cells, which occurred less rapidly compared to CD27^+^ B cell formation at hypoxic *p*O_2_ in line with time-dependent waves of memory B cell and antibody secreting cell formation *in vivo* (2). Finally, time-dependent transitions from hypoxic to normoxic *p*O_2_ during culture changes B cell differentiation trajectories and promotes IgG class switch recombination and Ig production. This indicates during *in vivo* B cell cycling between GC light zone and GC dark zone, the dynamic variation in *p*O_2_ is also likely to form another regulatory layer for human B cell differentiation. Overall, our results identify oxygen as a critical factor in dictating human B cell differentiation and demonstrate the necessity of incorporating GC-like physiological *p*O_2_ variations rather than continuous atmospheric *p*O_2_ to study human B cell responses *in vitro*.

*In vivo*, antigen-specific triggering of the BCR is required for the generation of an efficient GC response. We did not observe an effect on B cell survival or differentiation by addition of a BCR stimulus to the B cell cultures at different *p*O_2_, in line with previous reports showing limited additional effects of BCR triggers in *in vitro* cultures (12). Despite the absence of a BCR trigger, we saw a clear upregulation of c-Myc expression which is normally upregulated upon positive selection in the GC, in line with previous reports indicating CD40L ligation to be sufficient to induce c-Myc expression (25). We observed a reduction of c-Myc and NFκB expression in cells cultured at hypoxic *p*O_2_ after an initial surge upon culture initiation. c-Myc and NFκB signaling play a major role in transcriptional regulation of GC B cell proliferation and cell survival, thereby correlating with our observations regarding reduced cell numbers towards the end of cultures at hypoxic *p*O_2_. Naive B cell survival was abrogated upon culture at 0.5% *p*O_2_ and upon higher CD40L co-stimulation (subtype VH) at low *p*O_2_, but not atmospheric *p*O_2_, due to impaired proliferation. Hence, the level of CD40L co-stimulation impacts B cell survival at physiological *p*O_2_.

Highly proliferative lymphocytes have been described to adopt glycolysis (49), which -although not comprehensively studied- is in line with the increase in glycolysis-associated metabolic traits observed in our cultures independent of *in vitro p*O_2_. B cells cultured under hypoxic *p*O_2_ carried out mitochondrial oxidative metabolism, in addition to glycolysis. The difference in B cell differentiation trajectories and expansion of the CD27^+^ compartment in hypoxic cultures may explain the expanded metabolic program towards mitochondrial oxidative metabolism at day 7. Much of the research on crosstalk between immune responses and *p*O_2_ has focused on the TF HIF1α. We observe upregulation of HIF1α expression throughout all culture conditions not only at hypoxic *p*O_2_. HIF1α can also be induced by signaling through CD40L, BCRs as well as TLR ligands, probably explaining the increase in HIF1α also in non-hypoxic cultures (50). Furthermore, a recent study could not identify expression of hypoxia-induced genes in *ex vivo* analyzed mouse GC B cells (40), indicating HIF1α is not the sole regulator of hypoxia-associated responses.

Clear differences were observed in the expansion of CD27^+^ B cell and/or antibody secreting cell compartments in human naive B cells cultures at hypoxic and normoxic *p*O_2_, respectively, driven by differences in the underlying molecular pathways regulating B cell survival and differentiation. It has been shown that there is a time-dependent developmental switch in the output of the GCs, such that it first dedicates itself to memory B cell generation and later switches to mainly producing antibody secreting cells (2). During culture of naive B cells at hypoxic *p*O_2_ a hypoxia-dependent CD27^++^ population emerged early during culture. The question arises whether these CD27^++^ cells may resemble memory B cell generated early within a GC response. Albeit unanswered in this study, it did become clear that these CD27^++^ cells did not secrete Igs, whereas upon restimulation they swiftly differentiated into antibody secreting cells and exhibited efficient Ig secretion, in line with functionalities of memory B cells (51). Nevertheless, these cells clearly expressed a transcription factor signature of increased BLIMP1 and XBP-1s expression concomitant with a reduction of the B cell identity TF PAX5, similar to antibody secreting cells and different from memory B cells (16, 19). In line with varying *p*O_2_ during a GC response, where B cells cycle between hypoxic light zone to normoxic dark zone, we observed time-dependent effects of *p*O_2_ transitions on human B cell differentiation trajectories. Taken together, hypoxic *p*O_2_ primes B cells for efficient antibody secreting cell differentiation from a CD27^+^ phenotype that, in order to occur, favors a transition to a normoxic environment. Besides B cell differentiation trajectories, also IgG class switch recombination was influenced by *p*O_2_ transitions. When cultured at continuous *p*O_2_, atmospheric *p*O_2_ yielded the highest proportion of IgG^+^ B cells (albeit not corroborated with equally elevated levels of IgG antibody production). Similar numbers of IgG^+^ B cells could be generated in cultures that transitioned from hypoxic to normoxic *p*O_2_ at day 3 or 5. Overall, our *in vitro* studies demonstrate the necessity of incorporating GC-like physiological *p*O_2_ variations rather than continuous atmospheric *p*O_2_ to study human B cell responses *in vitro*, as it more closely resembles the *in vivo* GC microenvironment and recapitulates time-dependent regulation of B cell fate decisions.

Besides local *p*O_2_, other factors such as availability of nutrients, migratory and physical signals such as shear stress are also likely to contribute to B cell fate decision during both extrafollicular and GC B cell responses, warranting the need for 3D culture systems or organoids that more closely resemble the GC microenvironment to facilitate human *in vitro* B cell differentiation studies. In addition, several pathological conditions have been described to be related to hypoxia, such as myocardial ischemia (52), metabolic diseases (53), chronic heart and kidney diseases (54), and reproductive diseases (55). Moreover, *p*O_2_ has been measured and found to be significantly reduced in the majority of tumors, and sensitivity to chemotherapeutic agents changed dramatically under hypoxic conditions (56–58), highlighting the importance to adopt hypoxic cultures also for other avenues such as cancer-related drug testing and *in vitro* disease models.

In summary, we demonstrate that oxygen is an important regulator of human naive B cell differentiation by promoting oxygen-dependent differentiation trajectories and IgG class switch recombination.

### Materials and methods

#### Isolation of human naive B cells

Buffy coats were obtained from anonymized healthy donors with written informed consent in accordance to the guidelines established by the Sanquin Medical Ethical Committee and in line with the Declaration of Helsinki. Human Peripheral blood mononucleated cells (PBMCs) were isolated from fresh buffy coats using Ficoll gradient centrifugation (lymphoprep; Axis-Shield PoC AS). CD19^+^ cells were isolated by positive selection using magnetic Dynabeads (Invitrogen). Cryopreserved CD19^+^ cells were resuspended in PBA (PBS supplemented with 0.1% bovine serum albumin) and stained for surface markers 30 minutes in the dark at 4°C. Viable, singlet naive B cells (CD19^+^IgD^+^ CD27^-^IgG^-^IgA^-^) were sorted on a FACS Aria II/III.

### Human B cell cultures

3T3 mouse fibroblast cells expressing human CD40L (subtype: high), described previously (12), were harvested and irradiated with 30 Gy and seeded 10x10^3^ cells/well on 96-well plates for overnight adherence in B cell culture medium (RPMI medium supplemented with FCS (5%, Bodinco), penicillin (100 U/mL, Invitrogen), streptomycin (100 μg/mL, Invitrogen), β-mercaptoethanol (50 μM, Sigma-Aldrich), L-glutamine (2mM, Invitrogen), human apo-transferrin (20 μg/mL, Sigma-Aldrich) depleted for IgG using protein A sepharose (GE Healthcare). The next day, naive B cells were sorted and 250 naive B cells/well were co-cultured with the seeded 3T3-CD40L expressing cells in the presence of IL-4 (25ng/ml; Peprotech) and IL-21 (50ng/ml; Peprotech) for 3 – 11 days to determine proliferation, isotype switching, GC, memory and plasma cell formation and Ig production. Cultures were maintained at 37°C in an atmosphere with 5% pCO_2_ and 21%, 3% or 1% *p*O_2_. For some cultures after 3, 5 or 7 days cultures were moved from 1 to 3% *p*O_2_ and vice versa.

### Flow cytometry

#### Extracellular staining of surface markers

Cultured cells were washed with PBA and extracellular staining was performed at 4°C for 30 minutes using the following antibody conjugates; CD19 (562947), CD38 (646851), IgD (561315), and IgM (562977), CD138 (552723), CD21 (561372 or 740395), FAS/CD95 (762346), CD80 (750440 or 558226), CD86 (748375 or 562433), CXCR4 (563924) from BD Biosciences. IgG (M1268) from Sanquin Reagents. IgA (2050-09) from SouthernBiotech. CD27 (25-0279-42) from ThermoFisher. PNA (FL-1071-5) from VectorLabs. Glut1 (FAB1418A) from R&D. LIVE/DEAD Fixable Near-IR from Invitrogen.

#### Intracellular staining of signaling and transcription factors

Staining procedures were performed as previously described (59). In short, harvested and pooled cultures were kept on melting ice at all times. After washing, cultures were stained for membrane markers in a 25µl staining mix containing the Live/Dead stain, anti-CD19 and anti-CD38 antibodies. TF stain procedure also contained anti-CD27 antibodies during membrane marker staining. After staining, samples were washed once with ice-cold PBA and centrifuged. Samples were fixed with either paraformaldehyde (PFA; Sigma) or Foxp3 fixation buffer (eBioscience). PFA fixed samples were subsequently washed once and permeabilized with 90% methanol. Samples were incubated at -20 **°** C till day of FACS analysis. Foxp3-fixed samples were washed once with Foxp3 permeabilization buffer and kept in ice-cold PBA in the dark till day of FACS analysis.

Before FACS analysis, all samples were retrieved from -20 ^0^C or 4 ^0^C storage and washed once with ice-cold PBA. Methanol permeabilized samples were washed once more before adding 25µl staining mix containing antibodies against STAT3 (564133, BD), pSTAT3 (612569, BD), NFκB p65 (565446, BD), STAT6 (IC2167T, R&D), pSTAT6 (612600, BD) and C-MYC (13871S, CST), NFκB p65, HIF1α (359706, Biolegend) diluted in PBA. Samples were incubated for 45 minutes on a plate shaker at room temperature. Afterwards, samples were washed once with PBA and measured on a flow cytometer.

Foxp3 fixed samples were washed once with Foxp3 permeabilization buffer. Samples were stained in 25µl staining mix containing antibodies against PAX5 (649708, Biolegend), BCL6 (358512, Biolegend), IRF4 (646416, Biolegend) and AID (565785, BD) or PAX5, BCL6, IRF4, BLIMP1 (IC36081R-025, R&D) and XBP-1s (562820, BD) diluted in Foxp3 permeabilization buffer and incubated for 30 minutes in the fridge. Samples were washed once with Foxp3 permeabilization buffer and measured on a flow cytometer.

All antibodies used have been tested prior to experiments in target positive and negative cell lines/PMBCs/total CD19+ cells and were diluted to optimal staining concentrations. For overall population shifts the gMFI was plotted and expression frequencies were plotted when there was a particular expansion of high- or low-expressing cells. Samples were measured on the BD LSR Fortessa or BD FACSymphony and analyzed using Flowjo V10.8. For representative gating strategy, see **Figure S1**.

#### Proliferation assays

B cells were labelled with proliferation dye according to manufacturer’s instructions. In short, sorted B cells were washed with 10 ml PBS twice and resuspended to a concentration of 2x10^7^ cells/ml in PBS. Cells and 4µM Violet Proliferation Dye 450 (VPD450, BD Biosciences) in PBS were mixed at a 1:1 ratio and incubated 15 minutes in a 37°C water bath in the dark, vortexing the tube every 5 minutes to ensure uniform staining. Cells were washed twice using a 10 times volume of cold culture medium to end labeling. Thereafter, B cells were cultured according to the protocol described above.

#### Assessment of B cell metabolic status

To monitor B cell metabolic activity, cells were cultured as described and loaded with 50nM MitoTracker Red CMXRos (M7512, Invitrogen) and 25nM MitoTracker Green FM (M7514, Invitrogen), 20nM BODIPY™ FL C12 (4,4-Difluoro-5,7-Dimethyl-4-Bora-3a,4a-Diaza-s-Indacene-3-Dodecanoic Acid) (D3822, Invitrogen) or 200µM 2-NBDG (2-(N-(7-Nitrobenz-2-oxa-1,3-diazol-4-yl)Amino)-2-Deoxyglucose) (N13195, Invitrogen) for 30 minutes or 2.5uM MitoSOX (M36008, Invitrogen) for 10 minutes at 37 °CC 5% CO_2_ in pre-warmed Hank’s Buffered Salt Solution (HBSS, 14025050, Gibco) on day 0 and 7 of culture. Cells were washed, stained for membrane markers as described above and measured on a flow cytometer. Within experiments, for each sample, cell size was determined using FSC/SCC and gMFI was corrected subsequently according to the average cell size.

#### Cell counts

Before FACS analysis samples were mixed with at least 10.000 CountBright Absolute counting beads (Thermo Fisher Scientific) and prepared for flow cytometry analysis as described above. Absolute B cell counts were determined according to the formula:


$$\frac{\#LiveCD19+}{\#Beadsmeasured}x\#beadsadded$$

### Ig ELISAs of culture supernatants

IgM, IgA, IgG, and IgG1 and IgG4 expression levels were measured in culture supernatants at day 7, 11 and 14. 96-well maxisorb plates were coated with monoclonal mouse anti-human IgM (2μg/ml, MH15-1), anti-IgA (1μg/ml, MH14-01), anti-IgG (2μg/ml, MH16-1), anti-IgG1 (1μg/ml, MH161-01), and IgG4 (1μg/ml, MH164-01) in PBS all provided by Sanquin Reagents. Culture supernatants were incubated for 1h and secreted Igs were detected using 1μg/ml HRP-conjugated mouse anti-human IgM, IgA, IgG, IgG1, or IgG4 (Sanquin Reagents) in HPE (Sanquin Reagents). The ELISA was developed using TMB substrate, stopped by addition of 2M H_2_SO_4_ and absorbance was measured at 450 and 540 nm. OD values were normalized to values of a titration curve of a serum pool that was included in each plate.

### Lactate production

L(+)-lactate production was measured in B cell culture supernatant using L-lactate Assay Kit (ab65331, Abcam) according to manufacturer’s instructions. Lactate production was corrected for cell counts as measured by flow cytometry.

### Real-time semi-quantitative RT-PCR

RNA isolation was performed as described elsewhere (60). Primers were developed to span exon–intron junctions and then validated (IL-4R: 5’- CCCTGAAGTCTGGGATTTCCT -3’). Gene expression levels were measured in duplicate reactions for each sample in StepOnePlus (Applied Biosystems, Foster City, CA, USA) using the SYBR green method (Applied Biosystems, Foster City, CA, USA). Expression of housekeeping gene 16S was used for normalization.

### Statistical analysis

Statistical analysis was performed using GraphPad Prism 8. Data were analyzed using Repeated Measures one-way ANOVA with Tukey’s multiple comparison test, Repeated Measures two-way ANOVA with Sidak’s multiple comparison test or mixed-effects analysis with Dunnett’s multiple comparison test where appropriate. Results were considered significant at p <0.05. Significance was depicted as *p <0.05 or **p <0.01, ***p <0.001 or ****p <0.0001. To show significance between specific datasets within a graph containing multiple datasets, the * shows the significance between the dataset matching the color of * and the dataset closest to the *.

## Data availability statement

The original contributions presented in the study are included in the article/**Supplementary material**. Further inquiries can be directed to the corresponding author.

## Ethics statement

The studies involving human participants were reviewed and approved by Sanquin Medical Ethical Committee. The patients/participants provided their written informed consent to participate in this study.

## Author contributions

JK, CM, JS, ST, AB, SH, and TR designed research. JK, CM, JS, ST, and ND performed research. JK, CM and JS analyzed data. JK, CM, JS, AB, SH, and TR wrote the paper. All authors critically reviewed the manuscript, gave final approval of the version to be published, and agreed to be accountable for all aspects of the work ensuring that questions related to the accuracy or integrity of any part of the work are appropriately investigated and resolved.
