# Supplementary material for: Oxygen level is a critical regulator of human B cell differentiation and IgG class switch recombination
Source: Front Immunol. 2022 Dec 14;13:1082154. doi: 10.3389/fimmu.2022.1082154 (PMC9795029; doi:10.3389/fimmu.2022.1082154)
Supplement: Supplementary file 1 [file DataSheet_1.pdf]

Supplementary Figures

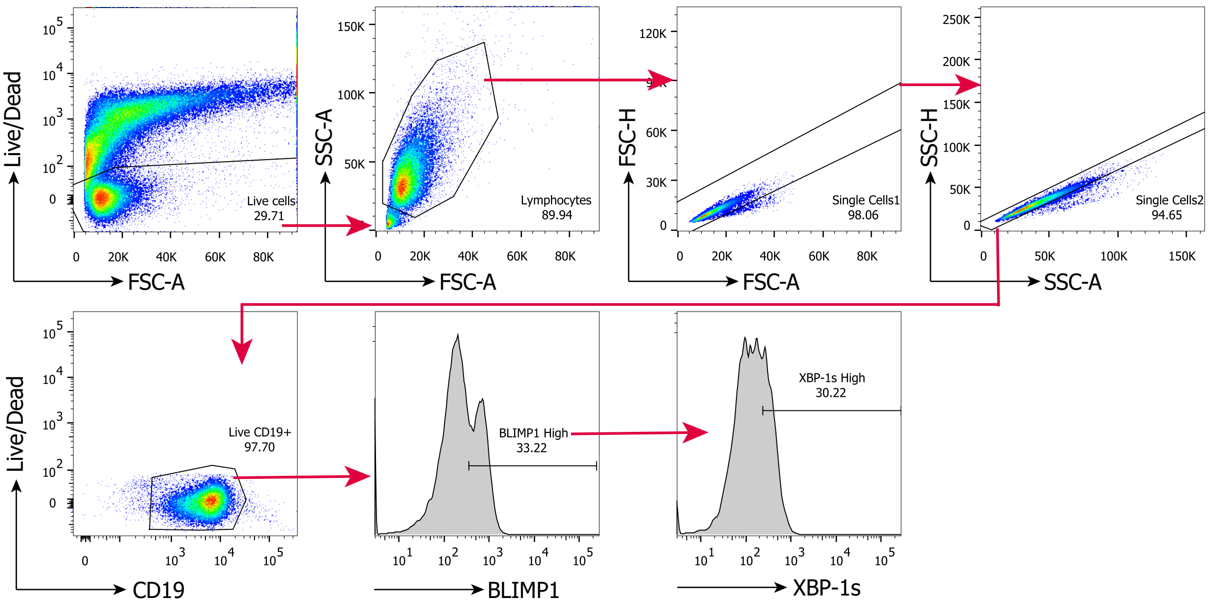

Figure S1. Repretentative gating strategy of B cells after culture. First live cells are gated based on viability dye and FSC-A, subsequently lymphocytes are gated using FSC-A and SSC-A, two gates to select singles, based on FSC and SSC A and H parameters. Finally cells were gated as live, CD19+ whereupon specific phenotyping and transcription factor markers were gated. Example gating strategy for BLIMP1 and XBP-1s gates.

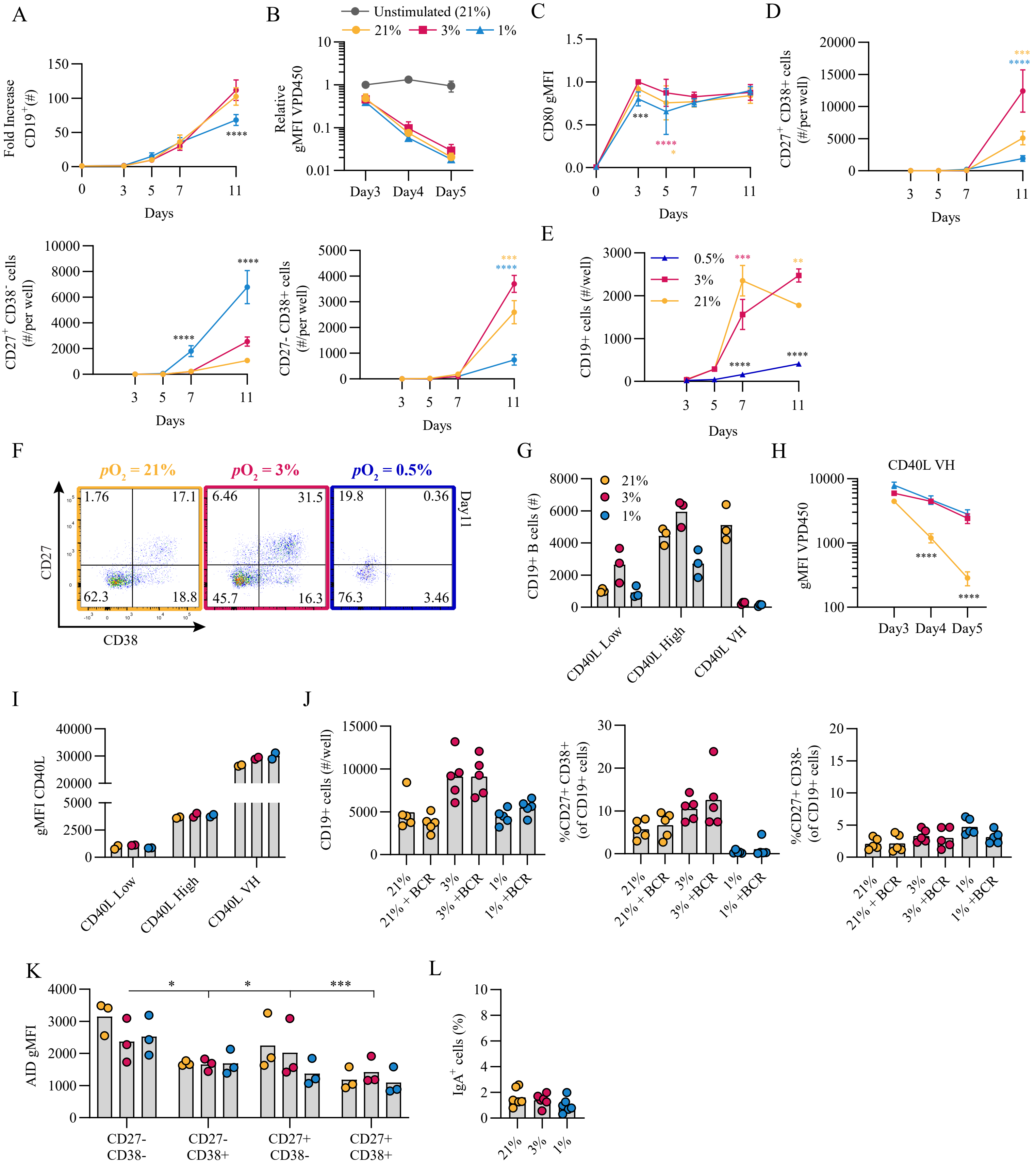

Figure S2. Hypoxia promotes human B cell differentiation *in vitro.* (**A**) Fold increase in number of CD19+ cells over the course of the culture compared to day 0 (250 cells/well, *n* = 9). (**B**) Proliferation (*n* = 3). (**C**) gMFI CD80 (*n* = 6). (**D**) The number of CD19+ cells retreived per well within the CD27/CD38 quadrants (*n* = 9). (**E**) Number of live CD19+ cells present in cultures using 0.5, 3 and 21 % pO_2 (*n* = 3). (**F**) Representative CD27/CD38 biaxial plot showing the differentiation of naive B cells at 0.5, 3 and 21% pO_2 at day 11 of culture. (**G**) Number of live CD19+ cells in cultures stimulated with IL-4 + IL-21 and human CD40L low, high or very high (VH) co-stimulation cultured at 21, 3 and 1% pO_2 for 11 days (*n* = 3). (**H**) As in (G) for proliferation at day 3, 4 and 5 (*n* = 3) (**I**) Expression levels of human CD40L low, high and very high (VH) expressed on mice 3T3 fibroblast cells cultured at 21, 3 and 1% pO_2 for 5 days (*n* = 2). (**J**) Number of live CD19+ cells, percentage of $CD27^+CD38^+$ and $CD27^+CD38^-$ cells in cultures with and without 1μg/ml anti-IgM F(ab')₂ cultured for 11 days. (*n* = 5) (**K**) AID expression within the different CD27/CD38 quadrants (*n* = 3). (**L**) IgA+ B cells after 11 days of culture (*n* = 6). Bars represent means of biological replicates each composed of two technical replicates of (**F-I, K**) 1, (**A, B, J, L**) 2 or (**C- E**) 3 independent experiments. Statistical differences were determined using (**B-F**) mixed effects model using Tukey's test for multiple comparisons. (**H-J**) repeated measures one-way

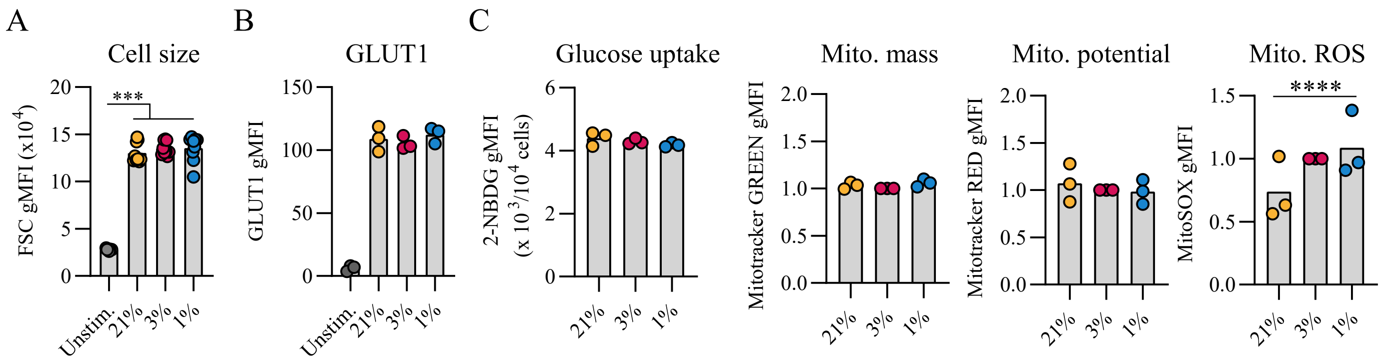

Figure S3. B cell cultured at hypoxic pO_2 alter their metabolic program. At day 7 of culture (**A**) Cell size determined as gMFI of the forward scatter (FSC, *n* =14), (**B**) gMFI of GLUT1 expression, and (**C**) gMFI of 2-NBDG, MitoTracker GREEN, MitoTracker RED, and MitoSOX in cells cultured at differential pO_2 for 11 days (*n* = 3). Bars represent means of biological replicates each composed of two technical replicates of (**A**) 2 (**B-C**) or 1 independent experiments. Statistical diferences were determined using repeated measures one-way ANOVA using Tukey's test for multiple comparisons. *** $p < 0.001$, **** $p < 0.0001$.

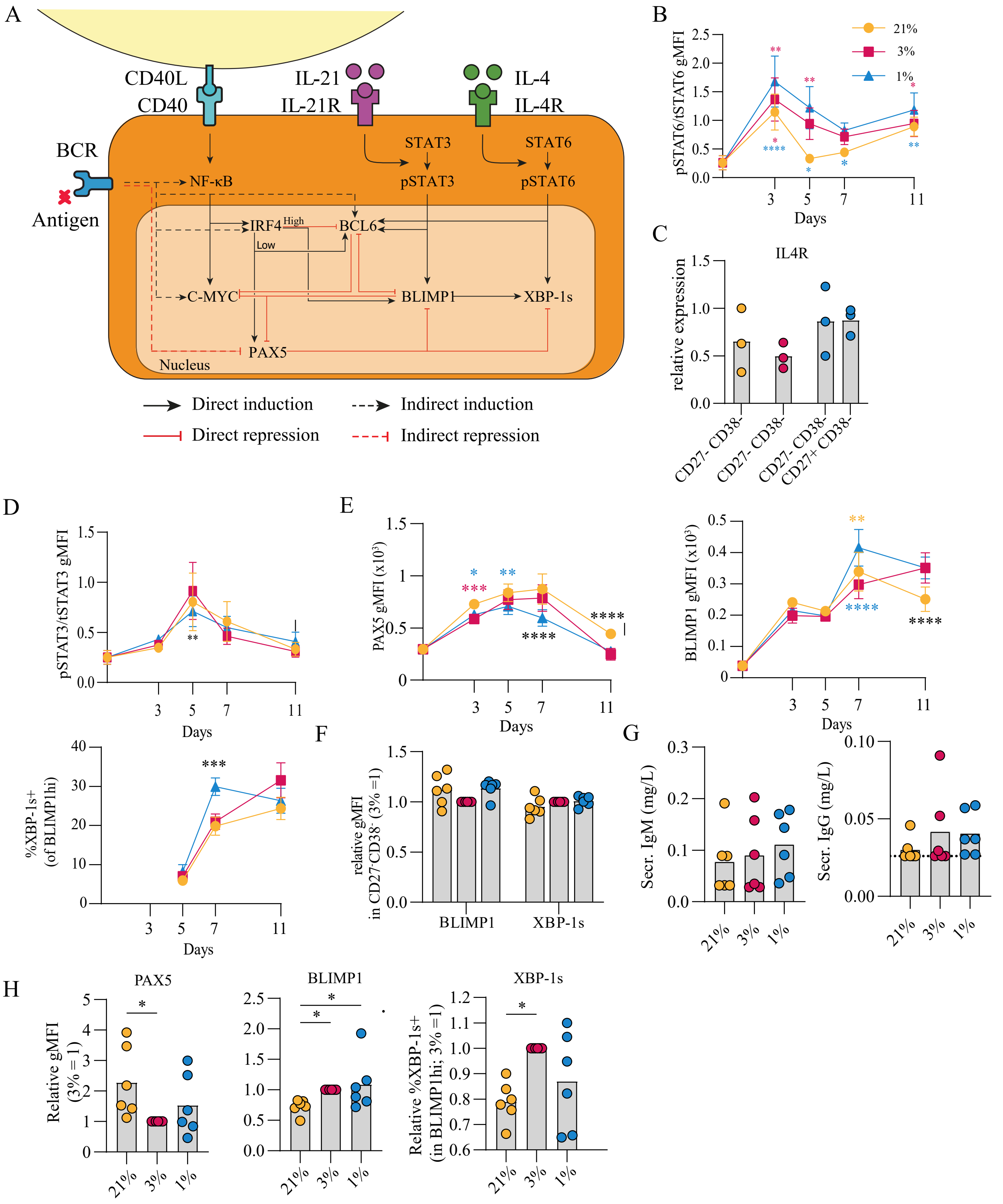

Figure S4 pO$_2$ steers molecular signaling underlying B cell differentiation (**A**) Schematic representation of intracellular signaling events leading to TF expression regulation upon B cell stimulation with typical Tfh signals including CD40L, IL-21 and IL-4. (**B**) ratio of pSTAT6/tSTAT6 gMFI (n = 6). (**C**) Relative expression of IL-4R mRNA as determined by RT-qPCR (n = 3). (**D**) ratio of pSTAT3/tSTAT3 gMFI (n = 6) (**E**) gMFI of PAX5, BLIMP1 and %XBP-1s over time (n = 6) (**F**) gMFI of BLIMP1 and XBP-1s within CD27$^-$CD38$^-$ population. (n = 6) (**G**) secreted IgM and IgG as measured by ELISA on day 7 of culture (n = 6) (**H**) gMFI of PAX5, BLIMP1 and %XBP-1s at day 11 (n = 6). Bars represent means of biological replicates each composed of two technical replicates of (**B, D-H**) 2 or (**C**) 1 independent experiments. Statistical differences were determined using (**B, D-E**) mixed-effects analysis using Tukey's test for multiple comparisons or (**C, F-H**) using repeated measures one-way ANOVA using Tukey's test for multiple comparisons.* $p < 0.05$, ** $p < 0.01$, *** $p < 0.001$, **** $p < 0.0001$.

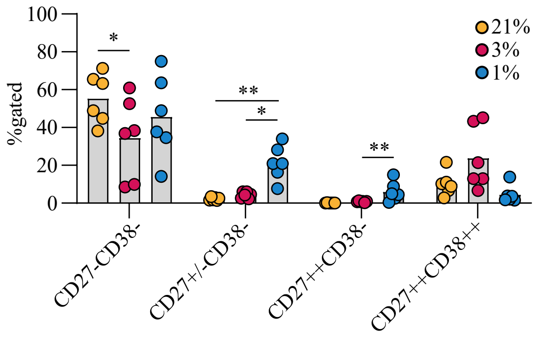

Figure S5 hypoxic *p*O_2 leads to generation of pre-ASC $CD27^{++}$ B cell subset. (**A**) Quantification of cells in $CD27^{-}CD38^{-}$, $CD27^{+/-}CD38^{-}$, $CD27^{++}CD38^{-}$, $CD27^{++}CD38^{++}$ gates on day 11 of culture (*n* = 6) (*n* = 5) Bars represent means of biological replicates each composed of two technical replicates of 1 independent experiment. Statistical differences were determined using mixed effects analysis using Tukey's test for multiple comparisons * p < 0.05, ** p < 0.01, *** p < 0.001, **** p < 0.0001

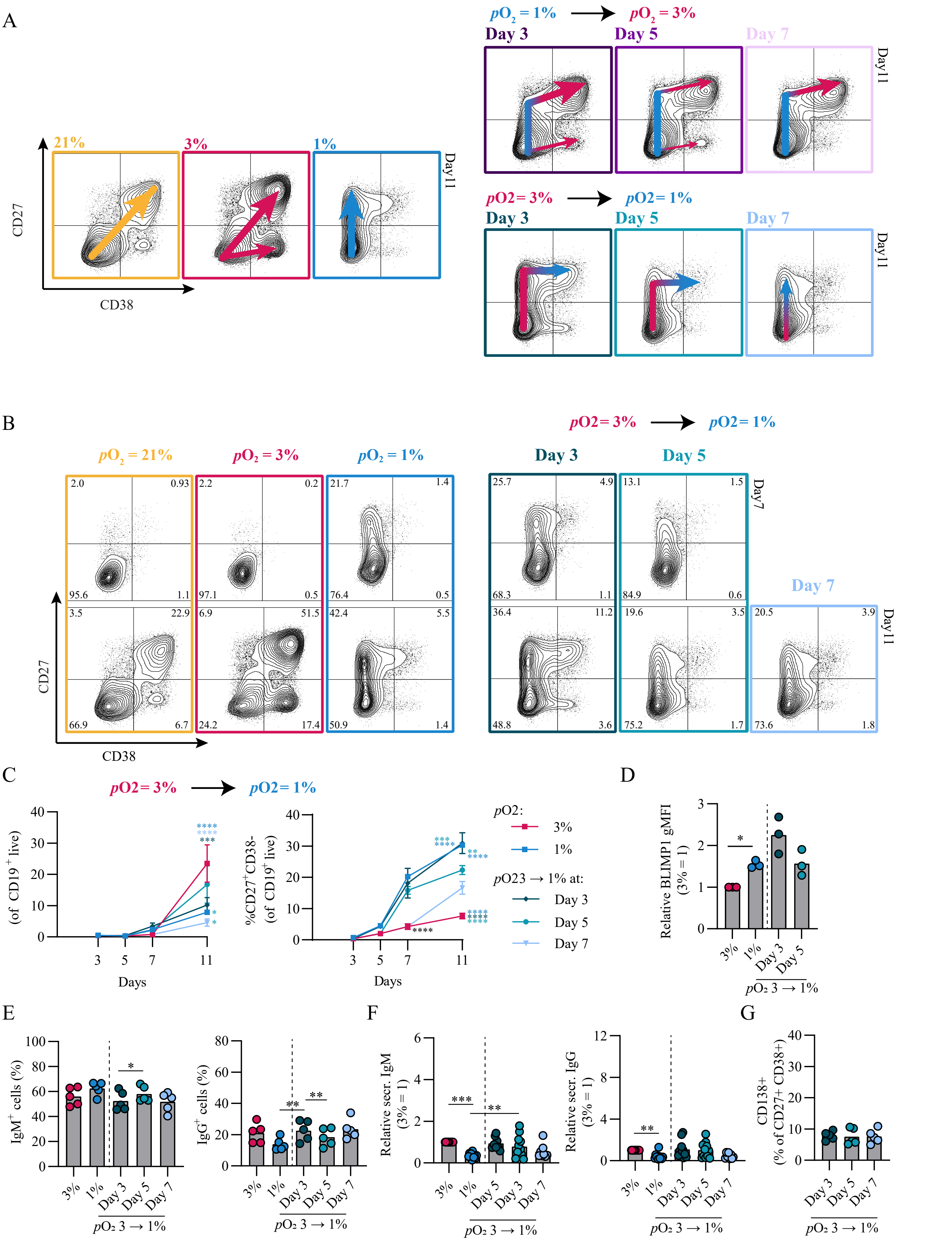

Figure S6. Time-dependent pO_2 transitions alter B cell differentiation dynamics and promote CSR to IgG. (**A**) Schematic representation of CD27/CD38 differentiation trajectories of human naive B cells *in vitro* (**B**) Representative biaxial CD27/CD38 FACS plots of 21, 3 and 1% pO_2 cultures and 3 - 1% pO_2 transition cultures at day 3, 5 and 7 shown for day 7 and 11 of culture. (**C**) Quantification of $CD27^+CD38^+$ and $CD27^+CD38^-$ B cell formation ($n = 11$) (**D**) gMFI of BLIMP1 at day 7 ($n = 3$) (**E**) Frequency of IgM^+ and IgG^+ cells determined by intracellular flow cytometry ($n = 5$) (**F**) and cumulative IgM and IgG secretion in culture supernatants at day 11 in 3 and 1% pO_2 cultures and 3 - 1% pO_2 transition cultures ($n = 11$). (**G**) CD138 expression within the $CD27^+CD38^+$ ASC population ($n = 5$). Bars represent means of biological replicates each composed of two technical replicates of (**C, F**) 3, (**E, G**) 2, or (**D**) 1 independent experiment. Statistical differences were determined using mixed-effects analysis using Tukey's test for multiple comparisons. * $p < 0.05$, ** $p < 0.01$, *** $p < 0.001$, **** $p < 0.0001$.
